# Estimating the burden of HIV late presentation and its attributable morbidity and mortality across Europe 2010–2016

**DOI:** 10.1186/s12879-020-05261-7

**Published:** 2020-10-07

**Authors:** The Late Presentation Working Groups in EuroSIDA and COHERE

**Affiliations:** https://www.eurosurveillance.org/

**Keywords:** HIV, AIDS, Late presentation, Avoidable events, Eastern Europe, HIV testing

## Abstract

**Background:**

Late presentation (LP), defined as a CD4 count < 350/mm^3^ or an AIDS-event at HIV-diagnosis, remains a significant problem across Europe. Linking cohort and surveillance data, we assessed the country-specific burden of LP during 2010–2016 and the occurrence of new AIDS events or deaths within 12 months of HIV-diagnosis believed to be attributable to LP.

**Methods:**

Country-specific percentages of LP and AIDS-events/death rates (assessed with Poisson regression) observed in The Collaboration of Observational HIV Epidemiological Research Europe (COHERE) and EuroSIDA cohorts, were applied to new HIV-diagnoses reported to the European Centre for Disease Prevention and Control. The estimated number of LP in the whole population was then calculated, as was the number of excess AIDS-events/deaths in the first 12 months following HIV-diagnosis assumed to be attributable to LP (difference in estimated events between LP and non-LP).

**Results:**

Thirty-nine thousand two hundred four persons were included from the COHERE and EuroSIDA cohorts, of whom 18,967 (48.4%; 95% Confidence Interval [CI] 47.9–48.9) were classified as LP, ranging from 36.9% in Estonia (95%CI 25.2–48.7) and Ukraine (95%CI 30.0–43.8) to 64.2% in Poland (95%CI 57.2–71.3). We estimated a total of > 320,000 LP and 12,050 new AIDS-events/deaths attributable to LP during 2010–2016, with the highest estimated numbers of LP and excess AIDS-events/deaths in Eastern Europe. Country-level estimates of excess events ranged from 17 AIDS-events/deaths (95%CI 0–533) in Denmark to 10,357 (95%CI 7768-147,448) in Russia.

**Conclusions:**

Across countries in Europe, the burden of LP was high, with the highest estimated number of LP and excess AIDS-events/deaths being in Eastern Europe. Effective strategies are needed to reduce LP and the attributable morbidity and mortality that could be potentially avoided.

## Background

Early HIV diagnosis and treatment are pivotal to maximizing individual and public health benefits of antiretroviral treatment (ART) [1, 2]. Despite increased focus on testing and linkage to care in recent years [3], across Europe, an estimated 40–60% of people do not present for HIV care until their CD4 cell count has decreased below 350 cells/mm3 or they have experienced an AIDS defining disease [4–6]. These late presenters [7] suffer individual health consequences in terms of high morbidity and mortality, especially during the first year after testing HIV-positive [8–10]. Additionally, late presentation (LP) has public health consequences in terms of increased risk of onward transmission of HIV and increased health care costs [11–13].

Many European countries have published data from cohort studies on prevalence and risk factors for LP [5, 8, 14, 15]. Less is known about the absolute numbers of patients with LP in individual countries, and excess morbidity and mortality that could be potentially avoided by earlier diagnosis and treatment. This is especially true for countries in Eastern Europe, where surveillance of HIV-infection may be more complex [16, 17].

The European Centre for Disease Prevention and Control (ECDC) publishes annual summaries of HIV across Europe, which may be more complete than data from individual cohorts, but are limited by a lack of information on outcomes after HIV-diagnosis and by incomplete reporting [15, 18, 19]. In contrast, large cohort studies, such as The Collaboration of Observational HIV Epidemiological Research Europe (COHERE) and EuroSIDA, include participants with HIV from many cohorts and countries, and collect information on the prevalence and outcomes of LP [20, 21]. Furthermore, the cohorts collect data from countries where surveillance data are limited. Linking surveillance data and cohort data, the aims of this study were therefore to estimate the burden of LP in 17 European countries, and to estimate the number of new AIDS-events or deaths in the 12 months following HIV-diagnosis that were believed to be attributable to LP.

## Methods

### Patients

We included participants from countries in the COHERE and EuroSIDA studies where at least 50 people presented for care during the study period. Regions were defined as in ECDC surveillance reports [15]. EuroSIDA holds the largest number of participants from Central (Poland) and Eastern European countries (Belarus, Estonia, Russia, Ukraine). Participants from these two regions were included from EuroSIDA and excluded from COHERE, to avoid duplicates. All people aged ≥ 16 years, who presented for care (defined as earliest date of HIV-diagnosis, first clinic visit, or enrolment into the participating cohort) for the first time after 1st January 2010, were eligible for inclusion. The last merger of COHERE data was in 2015 and therefore patients from COHERE were included to 31/12/2014. To increase the number of people included and thus ensure more precise estimates of LP in Central and Eastern Europe, we expanded the inclusion period to 01/01/2001–31/12/2016 for participants included from EuroSIDA.

Participants were required to have at least one CD4 cell count measured within 6 months of HIV-diagnosis and were excluded if date of HIV-diagnosis or sex was missing. People were also excluded if there was evidence of an earlier HIV-diagnosis (CD4 cell count, AIDS diagnosis, or having started ART) more than 1 month before first clinic visit. Persons from EuroSIDA were additionally excluded if they had tested HIV-positive more than 12 months prior to enrolment in the cohort, to reduce survival bias. Finally, within COHERE, persons from the seroconverter cohorts were excluded, as in previous LP-analyses [8].

### Definitions

Using the consensus definition, LP was defined as an HIV-diagnosis at a CD4 cell count below 350 cells/mm^3^ or with an AIDS-defining event regardless of CD4 count in the 6 months following HIV-diagnosis [7]. Events were classified as AIDS using the 1993 Centers for Disease Control and Prevention clinical definition. A new event after HIV-diagnosis was defined as either death or a new AIDS-event occurring more than 30 days after HIV-diagnosis or subsequent to the AIDS-event used to classify a person as LP.

### Statistical analyses

We used logistic regression to assess the change in LP over calendar time, adjusting for HIV transmission category, age, region of care, region of birth and delayed entry into care (> 3 months between HIV-diagnosis and first clinic visit). Multivariable linear regression was used to assess the change over time in CD4 cell count at HIV-diagnosis after adjustment for the variables listed above. Both analyses were performed separately for COHERE 2010–2014 and EuroSIDA 2001–2016. Estimates of the annual number of new HIV-diagnoses 2010–2016 were obtained from ECDC reports available online [15]. Estimates for Russia were also available online [22, 23].

We calculated the country-specific burden of LP, assuming that the percentages of LP observed in COHERE/EuroSIDA applied to all new HIV-diagnoses reported to ECDC/Russia, and that the rate of LP remained stable over the study period [5]. The 95% confidence interval (CI) for the percentage with LP was used to provide lower and upper bounds for these estimates.

We subsequently calculated the country-specific estimates of new AIDS-events/deaths within the first year following HIV-diagnosis that could be attributable to LP. First, the event rates in COHERE/EuroSIDA within 12 months of HIV-diagnosis were calculated separately for those with and without LP and were compared in Poisson regression adjusting for age and HIV transmission category. The mean follow-up time per country was also assessed. We then applied the country-specific rate of new AIDS-events/death of non-LP to the total reported number of persons diagnosed HIV-positive (from ECDC/Russia), assuming that the follow-up time within the first 12 months and the proportion of LP were the same as observed in the cohorts. The adjusted incidence rate ratio and 95% CI were applied to this number of non-LP to estimate the number of LP. The number of AIDS-events/deaths assumed to be attributable to LP, was calculated as the difference in event rates between LP and non-LP within each country. Belarus, Estonia, Poland, Russia and Ukraine reported no events in the first 12 months among non-LP, and for these countries, the average event rate and adjusted incidence rate ratio for the whole population (i.e. in all 17 countries) combined was assumed to apply. To account for differences in countries’ population size, excess events per 1 million inhabitants were calculated using population estimates from Eurostat [24].

### Sensitivity analyses

In sensitivity analyses, we assessed the generalisability of our estimates of LP and AIDS-events/deaths to all diagnosed HIV-positive in each country. Data from The European Surveillance System (TESSy) [25], provided by the ECDC, were used to weight cohort data to reproduce key demographic characteristics of all diagnosed HIV-positive in the study period. In brief, we compared key characteristics (age, sex and HIV transmission category) in our study population with TESSy data and assigned weights to either increase or decrease different demographic groups in our study [26]. For example, if 15% of participants in the UK were male, infected via injecting drug use and above 50 years, but this proportion using TESSy data was 20%, this group of individuals was under-represented in COHERE/EuroSIDA, and thus the number of LP or AIDS-events/deaths in this group was weighted upwards by 33% (0.2/0.15). Estimates from TESSy were not available for Estonia, Russia and Ukraine, and here we used the unweighted proportion estimated from COHERE/EuroSIDA. We repeated the analyses excluding events where AIDS occurred within the first 6 months, to reduce any potential bias from persons presenting with AIDS and being diagnosed with a new AIDS defining illness shortly thereafter.

## Results

Thirty-nine thousand two hundred four persons were included from the COHERE and EuroSIDA cohorts and overall, around half (*n* = 18,967, 48.4%; 95%CI 47.9–48.9) were classified as LP (Table 1). The majority lived in Western Europe (*n* = 38,511, 98.2%), were born in Europe (*n* = 25,746, 65.0%), were men who had sex with men (*n* = 20,061, 51.3%), had a median age of 37 years (interquartile range [IQR] 29–45) and CD4 cell count at diagnosis of 365 cells/mm^3^ (IQR 186–553). The majority of the LPs (77.5%) were classified as LP based on having a CD4 <  350 cells/mm^3^ (Table 2). The proportion with LP did not change significantly over time either in COHERE (2010–2014) or EuroSIDA (2001–2016). The adjusted odds of LP per later year of HIV diagnosis was 0.98 (95%CI 0.97–1.00 [*p* = 0.058]) in COHERE and 0.99 (95%CI 0.95–1.04 [*p* = 0.79]) in EuroSIDA. Likewise, the CD4 cell count at HIV-diagnosis did not change significantly over time: the change in CD4 cell count at diagnosis was 1.30 cells/mm^3^ per year (95%CI -0.80–3.40 [*p* = 0.23]) in COHERE and − 0.54/mm^3^ per year (95%CI -6.05–5.00 [*p* = 0.85]) in EuroSIDA.

**Table 1:** Characteristics of included patients from COHERE 2010–2014 and EuroSIDA 2001–2016

	All n (% of all)^a^	Late presenters n (% of LP)^b^
Total number of people included	39,204 (100)	18,967 (48.4)
Mode of infection		
Sex between men	20,061 (51.2)	7712 (38.4)
Sex between men and women - men	6262 (16.0)	3915 (62.5)
Sex between men and women - women	6400 (16.3)	3529 (55.1)
Injecting drug use - men	1293 (3.3)	753 (58.2)
Injecting drug use - men	401 (1.0)	165 (41.2)
Men other	3304 (8.4)	2006 (60.7)
Women other	1483 (3.8)	887 (59.8)
Region of residence		
West	38,511 (98.2)	18,629 (48.4)
Central (EuroSIDA only)	179 (0.5)	115 (64.2)
East (EuroSIDA only)	514 (1.3)	223 (43.4)
Region of birth		
Europe	25,746 (65.0)	11,773 (46.2)
Africa	3666 (9.4)	2345 (64.0)
Other	3670 (9.4)	2005 (54.6)
Unknown	6392 (16.3)	2844 (44.5)
Cohort		
COHERE 2010–2014	38,511 (98.2)	18,629 (48.4)
EuroSIDA 2001–2016	693 (1.8)	338 (48.8)
	**median (IQR)**	**median (IQR)**
Age (years)	37 (29–45)	39 (32–48)
CD4 cell count at entry into COHERE/EuroSIDA (/mm^3^)	365 (186–553)	180 (70–272)
Baseline† (month/year)	10/2011 (11/2010–11/2012)	09/2011 (11/2010–11/2012)

Countries included from EuroSIDA: Central Europe: Poland. Eastern Europe: Belarus, Estonia, Russia, Ukraine. † Baseline was defined as the earliest of positive HIV test, first study visit or cohort enrolment. ^a^percentage is a column percentage. ^b^percentage is a row percentage

**Table 2:** Estimated burden of late presentation by country and region 2010–2016

	Late presenters in COHERE/EuroSIDA		Total number diagnosed HIV-positive 2010–2016^a^	Estimated number of LP in the whole country 2010–2016
	Percentage with CD4 < 350/mm^3^ and/or AIDS	Percentage of LP with CD4 < 350/mm^3^ and no AIDS		
Country	% of all (95% CI)	% of LP	n	n (95% CI)
Austria	48.4 (45.1–51.6)	71.5	2200	1064 (993–1135)
Belarus	57.4 (47.5–67.4)	79.6	11,528	6622 (5470-7775)
Belgium	42.3 (38.8–45.8)	78.8	7675	3247 (2975-3519)
Denmark	52.8 (48.9–56.6)	74.3	1755	926 (858–994)
Estonia	36.9 (25.2–48.7)	100.0	2172	802 (547–1057)
France	48.6 (47.1–50.0)	76.6	41,148	19,979 (19,380-20,578)
Germany	56.8 (55.2–58.4)	66.1	22,186	12,604 (12,253-12,954)
Greece	47.5 (45.2–49.7)	86.6	5862	2782 (2648-2916)
Italy	52.0 (50.8–53.3)	81.9	26,691	13,882 (13,551-14,213)
The Netherlands	44.6 (43.2–46.0)	69.1	7206	3213 (3111-3316)
Poland	64.2 (57.2–71.3)	70.4	8268	5312 (4731-5892)
Russia	45.2 (37.7–52.8)	86.8	551,251	249,375 (207,886-290,865)
Spain	44.3 (43.2–45.5)	76.1	26,917	11,935 (11,624-12,245)
Sweden	56.7 (54.5–59.0)	81.1	3128	1774 (1703-1845)
Switzerland	53.0 (49.9–56.1)	79.8	3961	2100 (1977-2222)
United Kingdom	43.5 (42.1–44.8)	84.0	42,292	18,393 (17,824-18,963)
Ukraine	36.9 (30.0–43.8)	88.4	111,731	41,227 (33,500-48,954)
**Region**				
Central^b^	64.2 (57.2–71.3)		32,158	20,660 (18,402-22,918)
East^b^	47.1 (41.7–52.5)		786,651	370,472 (327,912-413,032)
West^b^	48.4 (47.9–48.9)		211,280	102,203 (101,148-103,257)

^a^As reported by ECDC, except for Russia, as described in text. ^b^As defined by ECDC. Regional estimates are not summations of those estimated for each country. *CI* Confidence Interval, *LP* Late Presentation

### Estimated burden of LP

Table 2 shows the percentage of LP within COHERE/EuroSIDA in each country where more than 50 study participants presented for care during the study period. The percentage of LP varied considerably from 36.9% in Estonia (95%CI 25.2–48.7) and Ukraine (95%CI 30.0–43.8) to 64.2% in Poland (95%CI 57.2–71.3). The estimated number of LP in the whole country in the period is also shown, assuming that the rates of LP observed in COHERE and EuroSIDA applied to all diagnosed HIV-positive in the whole country during 2010–2016. For example, in Russia, estimates suggest 551,251 persons were diagnosed HIV-positive during 2010–2016. If the rate of LP seen in this study (45.2%) applied throughout Russia, there would be a total of 249,375 LP over the same period (95%CI 207,886-290,865).

On the regional level, if the rates of LP observed in COHERE/EuroSIDA were assumed to apply throughout the region, we estimated that 102,203 people in Western Europe (95%CI 101,148-103,257), 20,660 in Central Europe (95%CI 18,402–22,918) and 370,472 (95%CI 327,912–413,032) in Eastern Europe would have presented late between 2010 and 2016 (Table 2).

### Estimated excess AIDS-events/deaths

The rates of new AIDS-events/deaths within 1 year following HIV-diagnosis, stratified by LP and non-LP are given in Table 3, in addition to the adjusted ratio between incidence rates in LP and non-LP. Overall, LP was associated with a 9-fold higher incidence of AIDS-events/deaths within 1 year of HIV-diagnosis (adjusted incidence rate ratio 9.3 [95%CI 7.2–12.0]), compared to non-LP. However, there was considerable heterogeneity between countries, ranging from a more than 15-fold higher incidence of new AIDS-events/death among LP in Belgium, Spain and France to a 4–5-fold difference in Denmark, Sweden, Switzerland and the UK. Some countries (Belarus, Estonia, Poland, Russia, Ukraine) had no new AIDS-events/death events reported among non-LP. For these countries, the average incidence (4.2 [95%CI 3.2–5.2]) as well as the average adjusted incidence rate ratio (9.3 [95%CI 7.2–11.9]) for the whole study population were assumed to apply.

**Table 3:** Estimated excess AIDS/death in the first year after HIV-diagnosis by country and region 2010–2016

			Non-late presenters	Late presenters	Difference LP versus non-LP	
Country	Number included from COHERE/EuroSIDA	Mean follow-up in COHERE/EuroSIDA in the first 12 months following HIV-diagnosis	1-year incidence of AIDS/deaths observed in COHERE/EuroSIDA^a^	1-year incidence of AIDS/deaths observed in COHERE/EuroSIDA^a^	Adjusted ratio of 1-year incidence of AIDS/deaths between LP and non-LP^b^	Estimated excess AIDS/deaths attributable to LP (difference LP – non-LP)^d^ 2010–2016
	n	mean PYFU	IR/1000 PYFU (95%CI)	IR/1000 PYFU (95%CI)	aIRR (95%CI)	n (95% CI)
Austria	920	0.89	4.7 (0.6–17.0)	63.3 (38.5–88.1)	10.20 (2.4–43.3)	44 (7–415)
Belarus^c^	94	0.98	4.2 (3.2–5.2)	37.7 (4.6–136.1)	9.3 (7.2–11.9)	169 (127–2754)
Belgium	747	0.84	2.8 (0.1–15.4)	79.4 (45.4–113.3)	25.4 (3.4–190.9)	250 (24–1152)
Denmark	635	0.83	7.9 (1.0–28.5)	61.8 (36.9–98.9)	4.2 (0.9–18.4)	17 (0–533)
Estonia^c^	65	0.98	4.2 (3.2–5.2)	86.6 (10.5–312.7)	9.3 (7.2–11.9)	47 (35–1768)
France	4531	0.68	2.5 (0.7–6.5)	59.8 (47.4–72.2)	19.2 (7.0–52.7)	661 (218–2581)
Germany	3774	0.83	4.4 (1.6–9.6)	58.6 (47.3–69.9)	11.1 (4.8–25.4)	353 (134–2408)
Greece	1829	0.82	5.0 (1.4–12.8)	43.0 (27.6–58.5)	7.5 (2.6–21.5)	82 (20–724)
Italy	6241	0.80	5.1 (2.5–8.8)	44.8 (36.7–53.0)	6.6 (3.6–11.9)	286 (134–2674)
The Netherlands	4680	0.84	5.4 (2.8–9.5)	69.8 (57.4–82.1)	9.9 (5.4–18.0)	163 (81–1490)
Poland^c^	179	0.99	4.2 (3.2–5.2)	8.8 (0.2–48.9)	9.3 (7.2–11.9)	102 (77–591)
Russia^c^	168	0.98	4.2 (3.2–5.2)	40.7 (8.4–118.9)	9.3 (7.2–11.9)	10,357 (7768-147,448)
Spain	7118	0.78	1.9 (0.7–4.2)	52.9 (43.8–62.0)	18.9 (8.3–43.2)	404 (165–1377)
Sweden	1830	0.81	7.9 (2.6–18.5)	32.7 (20.6–44.9)	3.7 (1.4–9.7)	24 (4–382)
Switzerland	998	0.82	5.4 (0.7–19.3)	25.0 (12.5–44.6)	5.2 (1.0–25.7)	34 (0–355)
United Kingdom	5208	0.72	5.4 (2.7–9.7)	37.6 (28.4–46.9)	5.1 (2.7–9.9)	382 (153–4252)
Ukraine^c^	187	0.98	4.2 (3.2–5.2)	59.2 (16.1–151.6)	9.3 (7.2–11.9)	2424 (1818-44,080)
**Region**^e^						
Central^c^	179	0.99	4.3 (3.3–5.4)	8.8 (0.2–48.9)	9.3 (7.2–11.9)	407 (306–2355)
East^c^	327	0.98	4.3 (3.3–5.4)	36.2 (18.7–63.2)	9.3 (7.2–11.9)	14,597 (10,947-109,680)
West	38,511	0.79	4.3 (3.3–5.4)	51.5 (47.9–55.2)	9.2 (7.2–11.9)	3030 (2273-19,934)

^a^Estimated number of events among LP or non-LP was estimated by applying event rates from COHERE/EuroSIDA to the total number diagnosed HIV-positive in the whole country (incidence rate (PYFU) x mean follow-up (PYFU) x number of LP or non-LP in the whole country). ^b^Adjusted for age, sex and HIV exposure group. ^c^In Belarus, Estonia, Poland, Russia and Ukraine there were no registered events among non-LP in EuroSIDA/COHERE. The IR and the aIRR given for these countries are the averages for all countries combined. ^d^Excess events were estimated as clinical events in LP compared to non-LP. ^e^As defined by ECDC. Regional estimates are not summations of those estimated for each country. *aIRR* Adjusted Incidence Rate Ratio, *CI* Confidence interval, *IR* Incidence Rate, *LP* Late Presentation, *PYFU* Person years of follow-up

Excess AIDS-events/deaths attributable to LP during 2010–2016 are also shown in Table 3, assuming that event rates among LP would be the same as in non-LP if they had been diagnosed and treated similarly. For example, if the event rate observed in the cohorts in the first year following diagnosis applied to all diagnosed HIV-positive in Austria, with a mean follow-up in the first year after HIV-diagnosis of 0.89 years, during 2010–2016 we would have expected to observe 49 AIDS-events/deaths in LP and 5 in non-LP in the first year after diagnosis, a difference of 44 events (95%CI 7–415). The highest burden of excess AIDS-events/deaths during 2010–2016 was expected in Russia (*n* = 10,357 [95%CI 7768–147,478]), and the lowest in Denmark, Sweden and Switzerland.

Table 3 also gives regional estimates, showing that the incidence of AIDS-events/death in non-LP in Western Europe, where there were most data, was 4.3/1000 PYFU (95%CI 3.3–5.4), and in LP was 51.5 (95%CI 47.9–55.2). Within Western Europe, the number of estimated excess AIDS-events/deaths in 2010–2016 attributable to LP was 3030 (95%CI 2273–19,934), in Central Europe 407 (95%CI 306–2355) and in Eastern Europe it was 14,597 (95%CI 10,947–109,680).

### Representativeness of EuroSIDA and COHERE

**Fig. 1:**
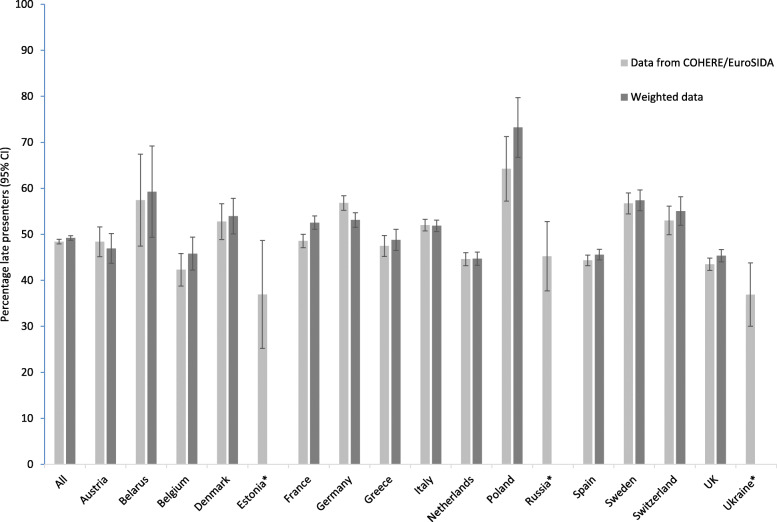
Proportion of late presenters in COHERE and EuroSIDA 2010–2016, using surveillance (TESSy) data as a reference population. Estimates were assigned weights to reproduce key demographic characteristics (age, sex, HIV transmission category) of all diagnosed HIV-positive in each country or region during the study period. * Not possible to calculate weighted estimate because of lack of data from either COHERE/EuroSIDA or TESSy. CI = confidence interval

When weights were applied to COHERE/EuroSIDA data to match the key demographic characteristics of the TESSy population, in general our data underestimated LP (Fig. 1). We could not include Estonia, Russia and Ukraine in this sensitivity analysis due to a lack of data from either COHERE/EuroSIDA or TESSy. The overall estimate of LP in the cohorts was 48.4% (95%CI 47.9–48.9), which increased slightly to 49.2% (95%CI 48.7–49.7), after accounting for differences in age, sex and HIV transmission category between COHERE/EuroSIDA and the TESSy reference population. Austria, Germany and Italy had a lower percentage of LP after weighting; all other countries had a higher percentage of LP after comparison with TESSy data, with the biggest increase in Poland (9.1%).

The weighted percentage of LP was then used to re-estimate the burden of LP and AIDS-events/deaths in the 12 months following HIV-diagnosis in 2010–2016. The estimated total number of LP rose marginally from 320,898 (95%CI 286,173-355,624) to 323,286 (95%CI 288,579-357,993), and excess AIDS-events/deaths among LP increased from 12,050 (95%CI 5064-18,348) to 12,164 (95%CI 5156-18,510) events.

### Effect of population size and new AIDS-events

**Fig. 2:**
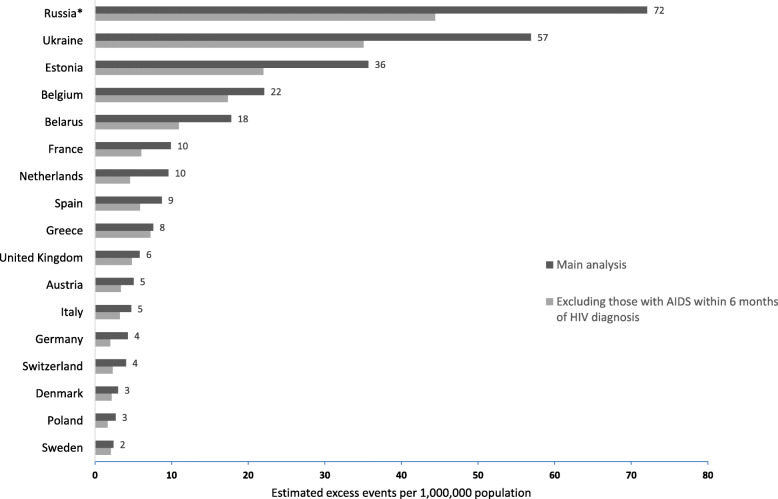
Estimated excess events per 1,000,000 inhabitants in each country. The figure shows the estimated excess AIDS-events/deaths attributable to late presentation, when differences in population size are accounted for. The sensitivity analysis gives the estimated excess events, when people with a new AIDS event within 6 months of HIV-diagnosis are excluded. The population estimates in each country are based on data from Eurostat (number of persons having their usual residence in a country on 1st January 2016) [24]. *For Russia, the population estimate from 2014 (most recent available) was used

As shown in Fig. 2, differences between countries remained when differences in population size were accounted for. For example, the highest number of excess AIDS-events/deaths attributable to LP during 2010–2016 was in Russia (72 events per 1 million inhabitants), and the lowest was in Sweden (2 events per 1 million inhabitants). Excluding those who developed AIDS within 6 months after HIV-diagnosis reduced the estimated excess events attributable to LP, but differences between countries remained, with the highest burden of excess morbidity and mortality observed in countries in Eastern Europe.

## Discussion

We aimed to quantify the burden of late presentation of HIV, and estimated that, among those diagnosed HIV-positive in 2010–2016, approximately half - equivalent to more than 320,000 people - were LP. We estimated that approximately 3030, 407 and 14,597 AIDS-events/deaths in Western, Central and Eastern Europe, respectively, were attributable to LP and could potentially have been avoided by earlier diagnosis and the concomitant potential for starting ART earlier. Our figures highlight that there is still a need to improve efforts to ensure timely diagnosis of HIV, and to reduce the health consequences of LP.

Since 2010 there have been many innovative approaches to increase and target HIV testing, including home testing, community-based testing, and indicator-guided testing [27–31]. Furthermore, an increase in the number of tests performed across Europe has been described [15]. Despite these initiatives, we found no evidence of a significant reduction in LP since 2010, which supports recent surveillance data that show no signs of decline in LP over the past decade [15]. Furthermore, the number of new HIV infections across Europe, in general, does not appear to be decreasing, and even shows signs of increasing among some subpopulations [32, 33]. Importantly, the steady increase in new infections in Eastern Europe is of particular concern [15, 34].

The pattern of LP varied across countries and regions. Reasons for LP are diverse, and include different testing strategies, challenges to identify and effectively reach those requiring a test, missed opportunities to offer a test, low self-perceived risk or limited knowledge about risk factors for HIV infection, fear of the implications of a positive test, perceived stigma and legal and regulatory barriers to testing and treatment [14, 27, 35–39]. There is a need for a comprehensive and holistic approach in order to address these known causes for LP. An additional barrier to timely treatment is delayed entry into care following diagnosis, although studies have shown that such delay accounted for less than 10% of LP [8, 36]. In our study the vast majority were classified as LP based on their CD4 cell count rather than an AIDS-event, which may indicate a need to increase testing based on indicator diseases as well as among individuals belonging to high prevalence populations. Furthermore, our estimates of the burden of LP were marginally higher when the representativeness of the data was accounted for, demonstrating that some populations may not be fully represented in our cohorts [26]. Our figures thus highlight the urgent need to more effectively target testing interventions to these populations before they present late.

This study extends our previous work by applying estimates of LP from cohorts to surveillance data, which may lack longitudinal follow-up and data on clinical outcomes. This allowed us to estimate the country-specific burden of LP and to provide estimates of excess morbidity and mortality occurring in the first year following HIV-diagnosis, attributable to LP. We estimate that during 2010–2016 a total 12,050 excess AIDS-events/deaths within the first year of HIV-diagnosis could potentially have been avoided if late presenters had been diagnosed with HIV and started treatment similar to those diagnosed early in COHERE and EuroSIDA. This likely represents an under-estimate of the clinical benefit of starting ART earlier. Our findings are based on cohort data in years when a large proportion of patients did not start ART until their CD4 cell count was below 350 cells/mm^3^, and largely before the results of the INSIGHT START study led to changes in clinical management in Europe [1]. This means that potentially even more events could have been avoided if all had been diagnosed and started treatment promptly [8, 10, 40].

We compared event rates among LP and non-LP followed at the same clinics within the same country. This allowed us to estimate potentially avoidable AIDS-events/deaths without taking into consideration variation in life-expectancy, health care organization, reporting practices and other factors that may affect between-country comparisons. We were, however, surprised that the observed event rates in countries in Eastern Europe were smaller than in some other countries [15, 41]. It is likely that individuals in our cohorts represent a subset of the whole HIV-diagnosed population with better outcomes. Participants in COHERE and EuroSIDA are engaged in clinical care, and we may therefore exclude people who are not under regular follow-up or those who do not survive long enough to be diagnosed and included in the cohorts. Furthermore, low event rates may cover incomplete reporting, despite our efforts to ensure high data quality. Also, we only considered new events within 12 months after HIV-diagnosis, and our analyses do not account for potentially avoidable events before or at HIV-diagnosis. Thus, our numbers probably underestimate the actual number of potentially avoidable AIDS-events/deaths.

A strength of this study is the country-level estimates of LP from seventeen countries across Europe, including countries in Eastern Europe where data are limited [42]. However, despite the large size of the COHERE and EuroSIDA cohorts, we were only able to include data from five countries outside Western Europe and we had comparatively small numbers from those countries. The limitations of extrapolating event rates from relatively few participants to the whole HIV-diagnosed population should be kept in mind when interpreting results. Further, for Russia there were no official statistics on the number of new HIV-diagnoses and we used estimates from sources available online. Increasing the availability and robustness of data from Eastern Europe remains an urgent priority.

For our main analysis, we assumed that persons in COHERE/EuroSIDA were representative of all HIV-diagnosed persons. In sensitivity analyses, we then used TESSy data to reproduce the demographic characteristics (age, sex and HIV transmission category) of the whole HIV-diagnosed population. Estimates were similar, but the burden of LP and excess AIDS-events/deaths attributable to LP were slightly higher using this weighted analysis. We chose not to use additional factors, such as ethnicity or CD4 count at presentation, because the sample size used for weighting within additional groups would have been small in some countries. Further, we were not able to use this sensitivity analysis in Estonia, Russia or Ukraine due to lack of data in COHERE/EuroSIDA and/or TESSy. Although our analyses indicate that our cohorts capture a representative sample of the whole HIV-diagnosed population, sites in EuroSIDA and COHERE are not selected at random and may not be representative of all clinics across the included countries. Finally, a limitation of our study is that different inclusion periods were used for COHERE (2010–2014) and EuroSIDA (2001–2016). This was done to increase the number of people included, and thus the robustness of estimates from Central and Eastern Europe. While the LP-rate has not changed significantly over the past decade, the awareness of timely ART, the components of ART-regimens and the overall quality of care may have improved over time. Thus, AIDS-events/death event rates from early in the study period may be higher than in recent years, which would tend to overestimate the number of potentially avoidable events.

The recent introduction of pre-exposure prophylaxis (PrEP) may be expected to reduce HIV transmission among some high-prevalence groups. However, we may speculate that the use of PrEP concentrates on people that have a high awareness of HIV and may be expected to test at a relatively early stage of infection. If so, PrEP may reduce HIV transmission, but not among those at highest risk for presenting late, thus leading to an overall increase in the percentage with LP in coming years.

## Conclusions

We found that LP remains a significant problem across Europe and translates into a substantial burden of morbidity and mortality, which could potentially be avoided by earlier diagnosis and treatment for HIV. An innovative and holistic approach spanning the HIV prevention, diagnosis, treatment and care continuum is needed to reduce the burden of HIV-related morbidity and mortality, which is potentially avoidable if people access timely ART.

## Data Availability

The EuroSIDA, COHERE and TESSy databases contain person-sensitive information and are therefore not publicly available, but are available from the corresponding author on reasonable request. *EuroSIDA.* The EuroSIDA Steering Committee encourages the submission of concepts for research proposals. Concepts can be submitted for review using an online research concept available at: https://chip.dk/Studies/EuroSIDA/Submit-research-concept. A submitted research concept will be evaluated by the Steering Committee for scientific relevance, design, feasibility and overlap with already approved projects. Upon completion of the review, feedback will be provided to the proposer(s). In some circumstances, a revision of the concept may be requested. If approved, a writing group will be established. Details on the EuroSIDA study can be accessed at https://chip.dk/Studies/EuroSIDA/About, where all relevant documents are available (study protocol, data collection, presentations, publications etc.). *COHERE.* The last merger of COHERE data was in 2015 and COHERE no longer collects new data. However, any researcher wishing to use COHERE data to conduct a project can download a COHERE Project Proposal Form and a Data Specification Form available at http://www.cohere.org/Projects. Proposals from external investigators will undergo the same rigorous scrutiny as those from investigators within the study group; details are outlined in the COHERE Manual of Operations at http://www.cohere.org/. *TESSy.* The European Surveillance System (TESSy) is coordinated by the European Centre for Disease Prevention and Control. Researchers may request to get access to subsets of data by contacting the TESSy data access team, as described at: https://www.ecdc.europa.eu/en/publications-data/european-surveillance-system-tessy. Access to aggregated published data is unrestricted and available at https://atlas.ecdc.europa.eu/public/index.aspx.

## Abbreviations

AIDS: Acquired immunodeficiency syndrome
ART: Antiretroviral therapy
CD4: CD4 receptor positive T-lymphocyte cell
CI: Confidence interval
COHERE: Collaboration of observational HIV epidemiological research Europe
ECDC: The European centre for disease prevention and control
EuroSIDA: Is not an acronym
HIV: Human immunodeficiency virus
IQR: Interquartile range
LP: Late presentation
PrEP: Pre-exposure prophylaxis
PYFU: Person years of follow-up
TESSy: The European Surveillance System
